# Evolutionary Insight into the Functional Amyloids of the Pseudomonads

**DOI:** 10.1371/journal.pone.0076630

**Published:** 2013-10-07

**Authors:** Morten S. Dueholm, Daniel Otzen, Per Halkjær Nielsen

**Affiliations:** 1 Department of Biotechnology, Chemistry and Environmental Engineering, Aalborg University, Aarhus, Denmark; 2 Interdisciplinary Nanoscience Center (iNANO), Centre for Insoluble Protein Structures (inSPIN), Department of Molecular Biology and Genetics, Aarhus University, Aarhus, Denmark; INRA Clermont-Ferrand Research Center, France

## Abstract

Functional bacterial amyloids (FuBA) are important components in many environmental biofilms where they provide structural integrity to the biofilm, mediate bacterial aggregation and may function as virulence factor by binding specifically to host cell molecules. A novel FuBA system, the Fap system, was previously characterized in the genus *Pseudomonas*, however, very little is known about the phylogenetic diversity of bacteria with the genetic capacity to apply this system. Studies of genomes and public metagenomes from a diverse range of habitats showed that the Fap system is restricted to only three classes in the phylum Proteobacteria, the Beta-, Gamma- and Deltaproteobacteria. The structural organization of the *fap* genes into a single *fapABCDEF* operon is well conserved with minor variations such as a frequent deletion of *fapA*. A high degree of variation was seen within the primary structure of the major Fap fibril monomers, FapC, whereas the minor monomers, FapB, showed less sequence variation. Comparison of phylogenetic trees based on Fap proteins and the 16S rRNA gene of the corresponding bacteria showed remarkably similar overall topology. This indicates, that horizontal gene transfer is an infrequent event in the evolution of the Fap system.

## Introduction

Functional bacterial amyloids (FuBA) represent an interesting class of very stable fibrillar protein polymers, in which the monomeric subunits fold as β-strands stacked perpendicular to the fibril axis [1,2]. Bacteria use FuBA for many purposes. The amyloids may act as simple structural proteins providing strength to biofilms and to the coating of spores. They may also have more specialized functions such as mediating specific binding to host cell proteins [3–5]. FuBA can be found within the extracellular polymeric substance (EPS) matrix of environmental biofilms from various habitats ranging from drinking water reservoirs and seawater to activated sludge from wastewater treatment plants [6,7]. FuBA are consequently considered important biofilm components.

It has previously been shown that many pseudomonads have the genetic capacity to express FuBA, the Fap system, as a part of their EPS matrix [8,9]. The species include the opportunistic pathogen *P. aeruginosa*, which is considered the major pathogen in cystic fibrosis airway infections, as *P. aeruginosa* colonization correlates with the onset of chronic pulmonary symptoms and declining lung function [10]. *P. aeruginosa* is furthermore a common inhabitant in chronic wounds [11]. *P. fluorescens* and *P. putida* also contain the Fap system and strains of these are known plant growth-promoting bacteria, interacting with plant roots through, among other factors, secreted proteins and biofilm formation [12,13]. Furthermore, strains of *P. putida* are prime candidates for bioremediation as they metabolize organic solvents and environmental toxins [14,15].

The Fap amyloid in *Pseudomonas* is expressed from a single operon, *fapABCDEF* [8,9]. FapC represents the major subunit of the mature Fap fibril, whereas FapB is a minor constituent. The exact function of FapB is unknown, but based on sequence similarity it has been suggested, that FapB might act as a nucleator analogue to CsgB in the *E. coli* curli amyloid system. Alternatively, it may be an integral part of the mature fibril, which could be used to modulate the physiochemical properties of the amyloid fibril. FapA affects the distribution of FapC and FapB in the mature Fap fibrils and is likely a chaperone for these monomers. Structural predictions suggest that FapD is likely a cysteine protease [9]. FapD could consequently have proteolytic activity relevant for processing the Fap proteins. Small amounts of FapE are found together with purified Fap fibrils. Accordingly, it could be hypothesized that FapE may represent an extracellular chaperone, guiding the fibrillation process. However, the exact function of FapE has to be confirmed. FapF is located in the outer membrane, where it according to structural predictions forms a β-barrel. It is a likely candidate for an outer-membrane pore for FapB and FapC secretion to the extracellular environment [9].

Recombinant overexpression of the *fap* operon results in highly aggregative phenotypes with enhanced biofilm forming capacity. The Fap fibrils are therefore considered structural components of *Pseudomonas* biofilms. However, Fap fibrils may also have more specialized functions. It is likely that *P. aeruginosa* utilizes Fap fibrils as virulence factors. This hypothesis is based on the identification of a *fapC* deletion mutant of *P. aeruginosa* as one of the most attenuated mutants, among 480 random transposon deletion mutants, in a *Caenorhabditis elegans* infection model and in a polymorphonuclear neutrophil leukocytes phagocytosis assay [16].

Although FuBA are abundant in nature, only a few FuBA systems have been identified so far and very little is known about the phylogenetic diversity of these systems. Consequently, it is not known whether the distribution of functional bacterial amyloids (FuBA) in natural systems result from a few phylogenetically widespread, but evolutionarily related FuBA systems, or from many independently evolved systems present within narrow groups of bacteria. Our investigations of the curli systems (Csg) showed that it was phylogenetically much more widespread then initially assumed, spanning at least four bacterial phyla [17]. Accordingly, this supports the idea that a few phylogenetic widespread FuBA systems could be responsible the high abundance of amyloids seen in environmental biofilms. However, insight from the phylogenetic diversity of a single FuBA system does not allow us to make any conclusions regarding this hypothesis. In order to gain a better understanding of the phylogenetic diversity of FuBA systems, we here present an investigation of the diversity of the Fap system.

We only found homologous Fap systems within species belonging to the Gamma-, Beta-, and the Deltaproteobacteria. Thus, these systems are much less widespread than the curli systems. The overall Fap operon structure is maintained between bacterial taxa in clear contrast to the instability seen for the curli operon. There is no evidence of horizontal gene transfer in the spreading of the Fap systems across genera. The Fap system may consequently be described as an evolutionarily young FuBA system compared to the curli system.

## Results

### Fap Genes are Phylogenetically Widespread

An initial identification of homologous Fap proteins within the refseq protein database was performed using PSI-Blast searches with the Fap proteins from *P. aeruginosa* PAO1 as query sequences (Table S1). The hits were manually curated and additional homologs were identified by manual examination of protein coding sequences (CDS) in the genomic neighborhood of the hits. The identified Fap homologs showed a high degree of variability in terms of primary structure. The average sequence identity between the Fap proteins from *P. aeruginosa* PAO1 and homologs outside the order of Pseudomonadales were 17% for FapA, 23% for FapB, 18% for FapC, 39% for FapD, 21% for FapE, and 32% for FapF.

Purely sequence-based methods, such as Blast searches, may not be able to detect evolutionarily related protein sequences if these have been subjected to intensive recombination and fast evolution [18]. The low sequence identity between Fap homologs suggests that divergence could provide a problem when searching for homologs. Profile hidden Markov models (HMMs) provide better detection of remote homologs than Blast searches [18,19]. The curated Fap homologs were consequently used to generate HMMs of the Fap proteins. As the putative nucleator protein FapB and the major Fap subunit FapC are internal homologs and these proteins are highly variable, a combined FapB/C HMM was constructed based exclusively on the repeat regions described by Dueholm et al. [8]. The HMMs were able to identify additional Fap homologs. The additional hits were curated as described earlier and the expanded Fap protein database was used to generate improved HMMs (Table 1 and HMMs S1). The iterative process was repeated until no additional homologs could be identified (Table S1).

**Table 1:** Validation of the Hidden Markov Models.

Target	HMM hits	Curated database	Correct hits	Missing hits
FapA	56	58	56/56 (100%)	2/58 (3%)
FapBC	138	135	135/138 (99%)	0/135 (0%)
FapD	1594	70	70/1594 (4%)	0/70 (0%)
FapE	71	69	69/71 (97%)	0/69 (0%)
FapF	217	69	68/217 (31%)	1/69 (1%)

The curated HMMs in general performed very well (Table 1). The FapB/C repeat model was able to detect 100% of all FapB/C proteins in our homolog database when applied to the refseq protein database. However, it also produced two false positive (corresponding to 1%). One of the false positives was found in the genome sequence of *Acidithiobacillus ferrivorans*. It contained two genuine Fap repeats and is very likely part of a partially deleted *fap* operon. The other false positive was a Bacteriodetes cell surface protein, which had five leucine rich repeat regions with some similarities to the Fap repeat regions. The curated FapA and FapE HMMs were very sensitive and highly specific. The FapA HMM did not include any false positives, making this model an excellent tool for the identification of novel Fap systems within metagenomes (see later). The FapE HMM was also able to identify all homologs, but also included two false positives. These false positives are very likely remains of partially deleted *fap* operons. One of the false positives was found in the genome sequence of the same strain of *A. ferrivorans* as the false positive FapB/C hit and the other was found in the *Burkholderia*, a bacterial family where the Fap system is frequently encountered. The curated FapD and FapF HMMs were both very sensitive but their specificity was very low, as seen by the high number of false positives, 96% and 69%, respectively. The non-specific hits likely indicate that FapD and FapF are part of two larger protein families.

**Figure 1:**
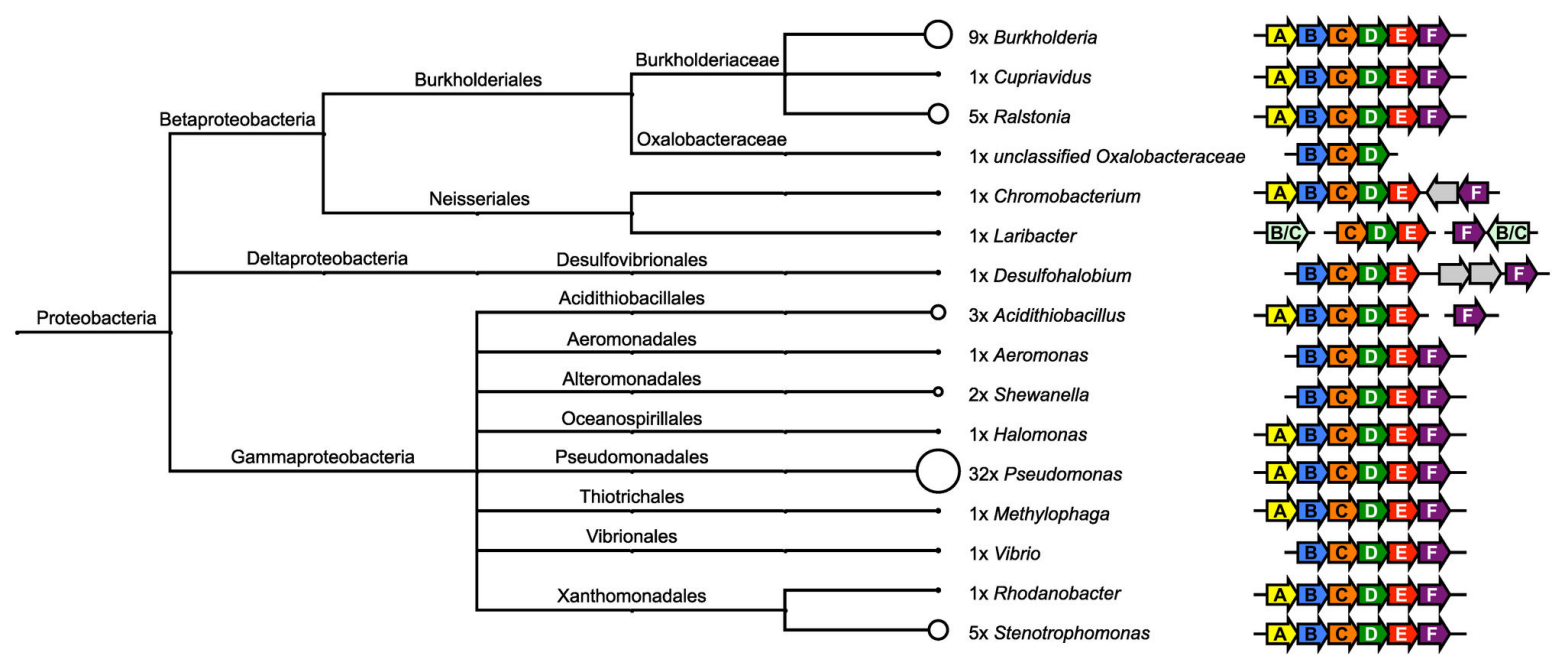
Phylogenetic Distribution of the Fap Systems and Operon Structure. Taxonomic analysis was performed based on the NCBI taxonomy and visualized using MEGAN [37]. The number of strains containing Fap systems within each genus is indicated next to the taxa and illustrated through the diameter of the circle. Note that these numbers are highly influenced by the number of sequenced strains within each phylogenetic group and do not reflect the prevalence of Fap systems within these groups. Organization of the *fap* operons is illustrated for each genus.

The combination of HMM searches and manual examination of the surrounding gene neighborhoods allowed identification of many novel Fap systems. They were all found within the Proteobacteria (Figure 1 and Table S1) and not in any other phylum with genomes available. The majority of the hits originated from the classes Beta- and Gammaproteobacteria, and a single system was identified within the genus *Desulfohalobium* of the Deltaproteobacteria. The Fap system could be found within 15% (4/60), 10% (9/91), and 4% (1/26) of the Beta-, Gamma-, and Deltaproteobacteria genera for with bacteria have been genome sequenced, respectively. This corresponds to 9.4%, 8.2%, and 0.7% of all genomes among the sequenced Beta-, Gamma-, and Deltaproteobacteria, respectively.

### Conservation and Organization of *fap* Genes

The arrangement into a single *fapABCDEF* operon was seen for all bacterial taxa, except for the genera *Chromobacterium* and *Laribacter* of the Betaproteobacteria, *Desulfohalobium* of the Deltaprotebacteria, and *Acidithiobacillus*, *Aeromonas*, *Shewanella*, and *Vibrio* of the Gammaproteobacteria (Figure 1). The most common deviations from the basis operon structure were the deletion of *fapA* and the separation of *fapF* from the main *fap* operon (Figure 1).

### Conservation of the Fap Fibril Monomers and Repeat Regions

**Figure 2:**
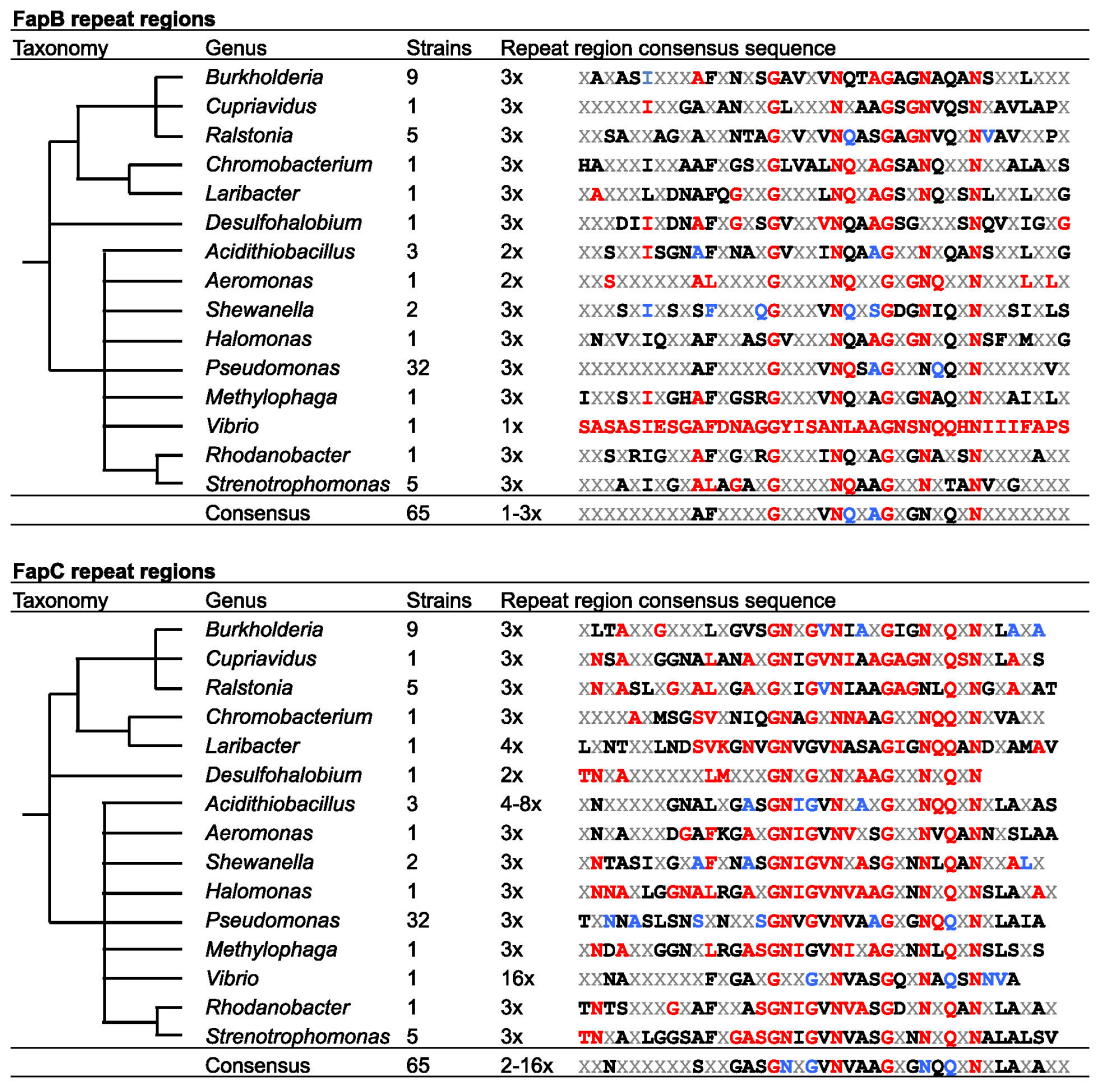
Comparison of Homologous FapB and FapC Repeat Regions. The phylogeny is based on the NCBI taxonomy. Bold residues represent 50% (black), 80% (blue) and 100% (red) conserved residues. Note that the conservation is highly biased by the number of sequenced strains and internal repeat units within the Fap proteins of each genus.

Homologs of the major Fap subunit FapC homologs had a similarly ordered primary structure composed of an N-terminal region, followed by three Fap repeats regions interspaced by linker regions and finally a C-terminal region. The FapC homologs displayed a large variation in size (377±143 (average ± standard deviation) amino acid residues). This variation in size is mainly due to highly variable linker regions. The variations from the common theme were FapC from *Desulfohalobium*, which lacks one repeat region, and the homolog from *Laribacter*, which contains an additional repeat (Figure 2). *Acidithiobacillus* and *Vibrio* stand out by having an even greater number (4-16) of FapC repeats.

FapB shared the same overall primary structure as FapC, but did not show the same variability in size (196±14 amino acid residues). The repeat regions were in most cases separated by linker regions of the same size. FapB from *Acidithiobacillus* and *Aeromonas* stand out by having only two repeat regions and only a single repeat region was seen for FapB in *Vibrio* (Figure 2).

A comparison of the FapB and FapC repeat regions from the different genera showed that only four out of 39 amino acids residues are fully conserved in the repeat regions of each protein. It is also interesting to notice the repeat regions of FapC were more conserved than those of FapB, having 56±25% and 45±26% average residue conservations, respectively.

### Evolution of Fap Systems

**Figure 3:**
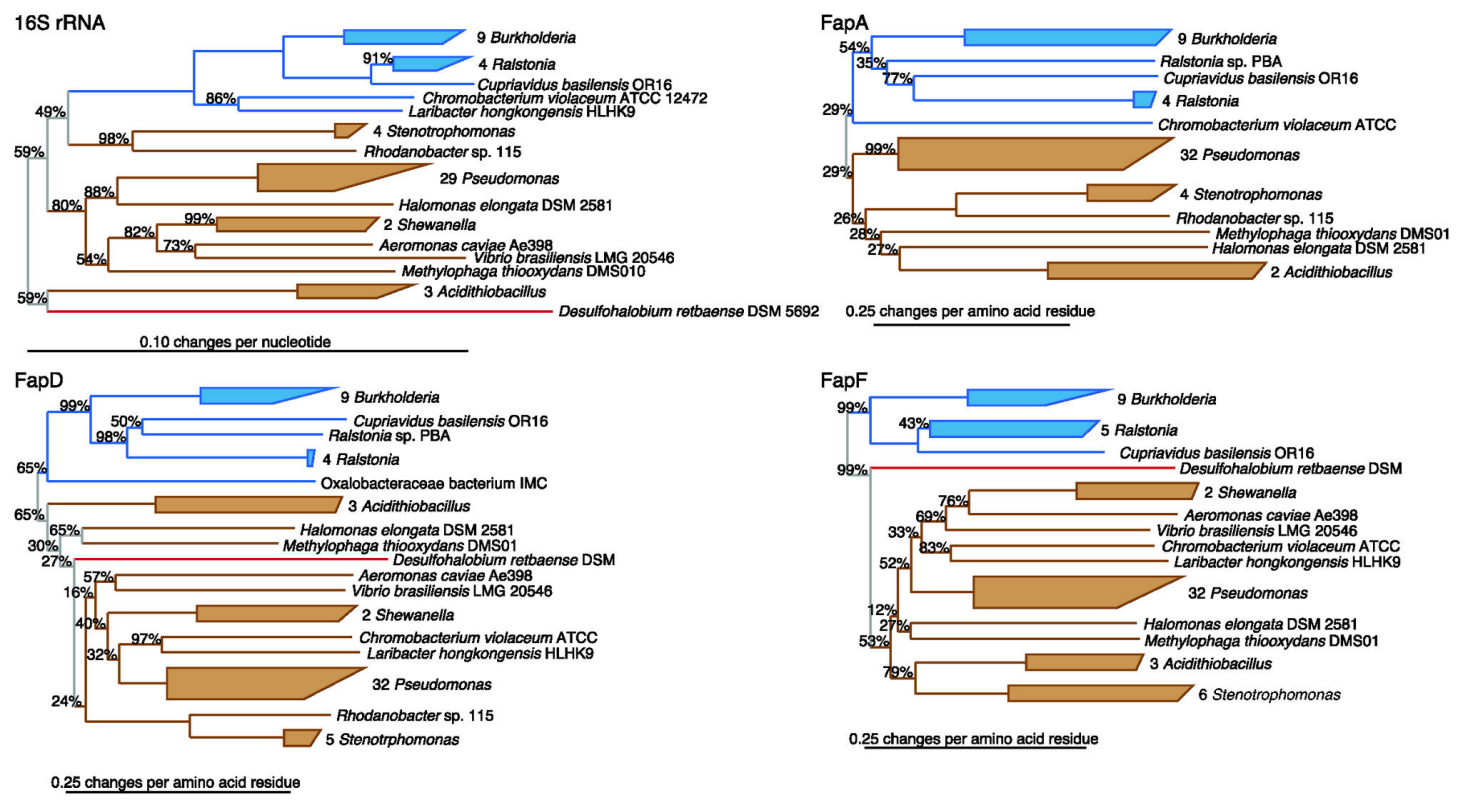
Evolution of Fap Systems. Comparison of phylogenetic trees based on the FapA, FapD and FapF protein sequences and corresponding 16S rRNA genes. The phylogenetic trees were estimated using the neighbor-joining method. Full-length 16S rRNA gene sequences could not be obtained for all Fap containing strains due to gaps in genome sequences. The phylogenetic tree is colored according to taxonomy. Betaproteobacteria (blue), Gammaproteobacteria (brown), and Deltaproteobacteria (red). Nonparametric bootstrap values are shown for each node. No bootstrap value represent 100% bootstrap support.

A comparison of phylogenetic trees based on functional genes or protein sequences with those of 16S rRNA gene sequences for the corresponding bacteria can be used to track the evolutionary history and allows differentiation between horizontal and vertical transmission of the functional genes. Phylogenetic trees based on the FapA, FapD, and FapF protein sequences and the 16S rRNA genes of the corresponding bacteria demonstrated remarkably similar overall topology (Figure 3). Fap homologs from all genera were localized in narrow genus-specific clusters. These observations imply that horizontal gene transfer was absent or only played a minor role in the spreading of the Fap system, and that the Fap systems might have evolved from a common ancestor. However, the low bootstrap support for the early branches makes it impossible to reliably determine the evolutionary origin of the Fap system.

### Fap Systems within Metagenomes

**Figure 4:**
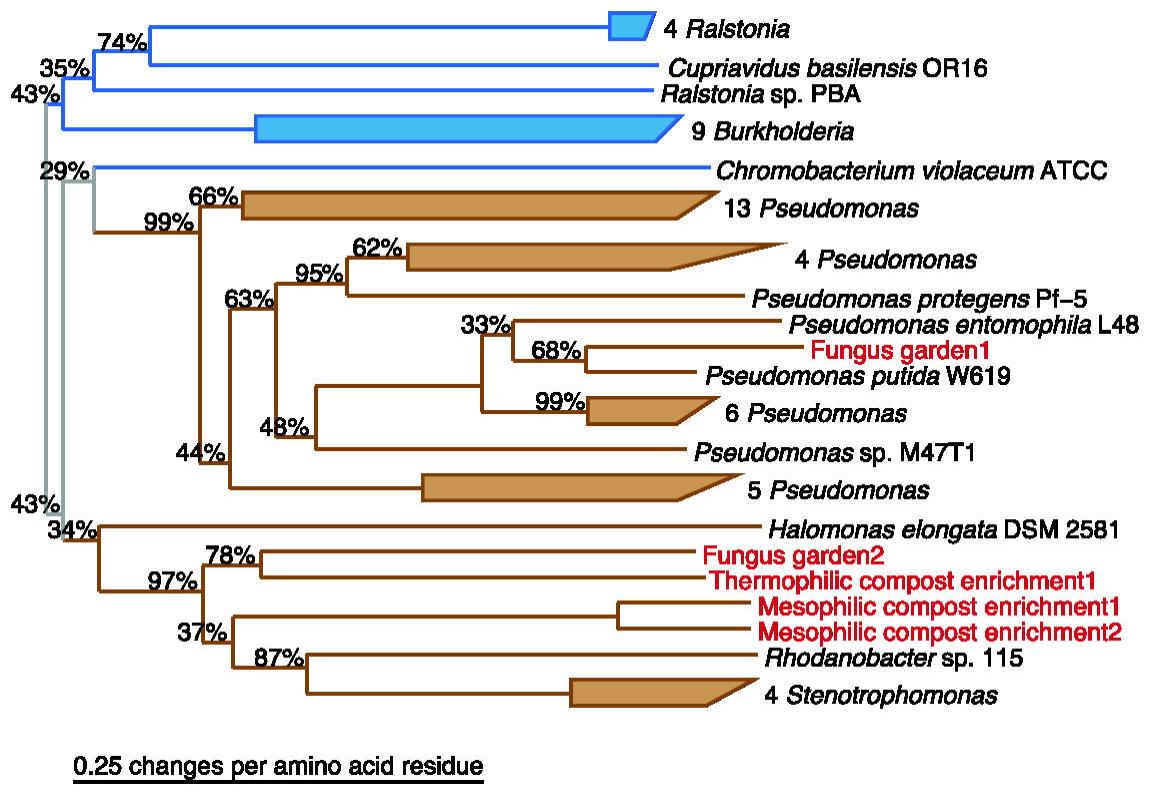
Fap Systems within Metagenomes. FapA homologs were identified using the respective HMMs within 10 large metagenomes from a diverse range of habitats, see Table 2. The hits were aligned with the FapA homologs identified within the refseq protein database and phylogenetic trees were estimated using the neighbor-joining method. The phylogenetic tree is colored according to taxonomy. Betaproteobacteria (blue), Gammaproteobacteria (brown), and Deltaproteobacteria (red). Metagenome hits are highlighted in red text. Nonparametric bootstrap values are shown for each node. The absence of a bootstrap value represent 100% bootstrap support.

The number of genome-sequenced bacteria has increased rapidly over the last couple of years. However, there is still a strong bias towards clinically relevant and cultivable bacterial strains. The highly specific FapA, FapB/C repeat, and FapE HMMs were therefore used to identify Fap systems within ten large metagenomes covering a broad range of habitats (Table 2). Fap systems could be identified within 50% of the metagenomes. The FapA hits were aligned with the FapA proteins identified within the Refseq protein database in order to produce a phylogenetic tree (Figure 4). We were not able to construct a similar tree for FapE due to the low quality of the FapE metagenome hits (the hits corresponded to protein fragments). The FapA hits were all found within the phylogenetic tree of the previously identified FapA homologs. One of the hits showed up within the genus *Pseudomonas*. The remaining four hits were related to the Xanthomonadales (*Rhodanobacter* and *Stenotrophomonas*), but the evolutionary distances suggest these hits likely originate from another bacterial order for which we still lack genome-sequenced strains with the *fap* operon.

**Table 2:** Fap systems within Metagenomes.

Metagenome name	Abbreviation	Size (proteins)	FapA	FapB/C	FapE	IMG/M taxon object id
Guaymas Basin hydrothermal plume	Hydrothermal plume	319,874	0	0	0	2061766003
Soil microbial communities from sample at FACE Site Metagenome	Soil	1,057,446	0	0	0	2124908009
Mesophilic rice straw/compost enrichment metagenome	Mesophilic compost enrichment	840,360	2	3	3	2199352012
Thermophilic rice straw/compost enrichment metagenome	Thermophilic compost enrichment	432,661	1	3	2	2199352008
Fresh water microbial communities from LaBonte Lake, Laramie, Wyoming, sample from algal/cyanobacterial bloom material peak-bloom 2	Fresh water	665,401	0	0	0	2189573023
Sediment microbial communities from Arctic Ocean, off the coast from Alaska, sample from low methane PC12-247-20cm	Artic ocean sediment	784,879	0	1	1	2100351001
Fungus garden microbial communities from Atta colombica in Panama, sample from dump	Fungus garden	1,285,907	2	9	2	2038011000
Svalbard Reindeer rumen metagenome	Reindeer rumen	813,781	0	0	0	2088090000
HumanGut BGI gene set	Human gut	3,064,560	0	0	0	4448044^1^
Aalborg West enhanced biological phosphor removal waste water treatment plant	Aalborg West EBPR	1,636,090	0	5	1	Not published

FapA, FapB/C and FapE homologs where identified using the developed HMMs.

^1^MG-RAST id

## Discussion

### Phylogenetic Diversity of the Fap System

The Fap system was phylogenetic restricted to three classes within the Proteobacteria, and does not match the phylogenetic diversity seen for the curli system, which spans at least four bacterial phyla [17]. The absence of noticeable horizontal gene transfer in the evolution of Fap system combined with the lower phylogenetic divergence implies that the Fap system is an evolutionarily young FuBA system compared to the curli system.

The identification of Fap systems within metagenomes from diverse habitats ranging from compost enrichments and fungus gardens to Artic Ocean sediments and wastewater treatment plants is in good agreement with the wide range of habitats covered by the bacteria containing the *fap* operon. It should be noticed that genera containing the *fap* operon involve both aerobes, such as *Pseudomonas* and strict anaerobes, such as *Desulfohalobium*. Fap producing bacteria may hence be encountered in most habitats.

### Fap Systems within Pathogenic Bacteria

Fap fibrils have been suggested to function as virulence factors for *P. aeruginosa*, as deletion of the *fapC* gene in a *P. aeruginosa* strain resulted in a highly attenuated phenotype in a polymorphonuclear neutrophil leukocytes phagocytosis assay [9,16]. It is therefore interesting to notice that several other bacteria containing the genetic elements to produce Fap are pathogens. They include *Aeromonas caviae* and *Laribacter hongkongensis*, which can both cause gastroenteritis and diarrhea [20,21]. Many of the other pathogenic bacteria identified are associated with opportunistic infections in the airway and lungs of patients suffering from cystic fibrosis and infections in immunocompromised individuals. These pathogenic species include *Burkholderia gladioli*, *B. pseudomallei*, *Ralstonia pikettii* and *Stenotrophomonas maltophilia* [22,23]. *Chromobacterium violaceum* is a rare pathogen, but it is associated with high mortality due to a rapid progress to sepsis with metastatic abscess in the liver, lung, or spleen [24]. The high frequency of known pathogen strains within the identified bacteria carrying the *fap* operon (39%) combined with results from the phagocytosis assay implies, that Fap might be involved in virulence. However, not all pathogenic species of the genera *Burkholderia* and *Stenotrophomonas* contain the genetic elements required for Fap expression. For example, the *B. cepacia complex*, which is a major pathogen of the *Burkholderia* genus, does not contain any *fap* genes. Fap is consequently not a requirement for pathogenicity, but may rather be a virulence enhancement factor. Additional experiments are required to confirm the virulence enhancing function of Fap for the pathogenic strains.

### Fap Systems within Plant Root-Associated Bacteria

The rhizosphere of plants provides a nutrient rich environment compared to the bulk soil, due to the release of root exudates. This ecological niche promotes the growth of mutualistic and commensal bacteria, which in turn provide the plant with protection against plant pathogens through the secretion of antibiotics and better access to nutrients via the secretion of hydrolytic exoenzymes [25,26]. It is now widely acknowledged that the ability to form biofilms is valuable if not a necessity for bacteria interacting with plant root [25,27]. The biofilm lifestyle provides the bacteria with a handful of advantages, such as increased resistance to environmental stresses and protection from protozoan grazing [28,29]. It furthermore provides the bacteria with an opportunity for metabolic cooperation as well as cross-feeding between different bacterial species residing in close proximity within microcolonies [30].

It is interesting to notice that many of the *fap* carrying bacteria identified in the study are known rhizobacteria (36%). These are mainly found within orders Burkholderiales, Pseudomonadales and Xanthomonadeles. Currently, it is not known whether these bacteria use Fap when they associate with plant roots. However, the fact that the Fap system in *P. aeruginosa* PAO1 has a higher expression at temperatures below 30°C and that Fap in general are highly potent biofilm mediators suggest that this might be the case [9]. The involvement of FuBA in plant root colonization has been confirmed for the curli system [31]. It was shown that the plant growth-promoting *Enterobacter cloacae GS1* expresses curli during root colonization and deletion of curli genes attenuated the plant growth promoting activity.

It has been suggested that certain bacteria, such as *S. maltophilia* may protect plants against nematode attacks, as clinical isolates of these bacteria are highly virulent in nematode killing assays [32]. High virulence was in a similar assay also observed for *P. aeruginosa*. However, in the latter experiment it was shown that deletion of *fapC*, coding for the major component of the Fap fibril, attenuated the virulence [16]. Fap may consequently play a role as a nematocidal agent in the rhizosphere.

### Conserved Operon Structure

The *fap* operons are remarkably conserved when compared to the unstable operons of the curli systems [17]. This is in line with an evolutionarily young FuBA system. However, there are some deviations from the common *fapABCDEF* theme. The most common deviation (6/16 genera) is an absence of *fapA*. Deletion of *fapA* has been shown to affect the distribution of FapB and FapC in the mature Fap fibrils in *Pseudomonas*, but it does not disrupt Fap dependent biofilm formation [9]. Strains that lack *fapA* may consequently produce functional Fap fibrils. Another common deviation (4/16 genera) is the separation of *fapF* from the main *fap* operon. Splitting the *fap* operon in two does not necessarily impair its function, but it requires the introduction of a new promoter region linked to *fapF*. A promoter region containing -35 and -10 regions could be identified in front of the lone *fapF* in *Chromobacterium* using the prokaryotic promoter prediction tool Softberry-BPROM (http://linux1.softberry.com). No promoter regions were found in front of the detached *fapF* within the other genera. However, the *fapF* genes of these genera were closely associated with upstream genes. It is therefore possible that *fapF* is integrated in a novel operon structure.

### Conservation of the Fap Fibril Subunits

A substantial variation in the primary structure and number of repeat units within the major curli subunit across taxa was previously reported [17]. The major Fap subunit also shows considerable variation in is primary structure, however the number of repeat units are highly conserved with the exception of *Laribacter, Desulfohalobium, Acidithiobacillus*, and *Vibrio*. The major variations between FapC homologs are seen in the linker regions separating the repeat units. The current model of the mature Fap fibril suggests that the repeat units make up the tight amyloid core, whereas the linker regions are exposed and in direct contact with the external environment [9]. If this is the case, the variation in the linker regions could be used to modulate the chemical properties of the Fap fibrils or mediate their binding to specific interaction partners.

A comparison of the FapC repeat regions from the different genera shows that only four amino acids residues (10%) are fully conserved. This is in the same range as for the curli repeats where three amino acid residues are fully conserved (14%) [17]. However, the average residue conservation of the Fap repeats across all homologs (47±24%) is higher than for the curli repeats, even if the latter is examined solely for the Enterobacteriales (42±30%). This is in good agreement with the idea that the linker regions determine the properties of different Fap homologs and the repeat regions constitute a common amyloid scaffold. As there are no linker regions in the curli subunits, the curli fibril properties have to be determined by the repeat unit sequences. The repeat units in CsgB are structurally better conserved than those in CsgA. The higher degree of repeat conservation within CsgB has been proposed to be due to the need for additional structural constraints within a nucleator protein [17]. The homology of FapB with FapC and the presence of small amount of FapB in mature fibril purifications suggests, that FapB is a putative nucleator protein in the Fap system analogous to CsgB in the curli system [8,9]. Alternatively, FapB could be an integral part of the mature fibril, which modulates the physiochemical properties of the mature Fap fibril. Surprisingly, the repeat regions of FapB are less conserved than those in FapC. However, the smaller size and the more conserved linker regions in FapB may provide the additional structural constraints. The more conserved repeat units in FapC might provide an additional stability to the amyloid core required to maintain the structure despite highly dynamic exposed linker regions.

### Increased Number of Repeat Units in *Acidithiobacillus* and *Vibrio*

The Fap systems of *Acidithiobacillus* and *Vibrio* provide some interesting deviations from the common Fap theme. FapB from *Acidithiobacillus* and *Vibrio* contains only two and one repeat unit, respectively. It may subsequently be suspected that these bacteria are not able to produce functional Fap, assuming a nucleator function of FapB. However, the FapC homologs of *Acidithiobacillus* and *Vibrio* contain 4-8 and 16 repeat units each. These bacteria may thus rely on self-nucleation by the repeat expansion within FapC instead of the proposed heterologous nucleation by FapB. This hypothesis is supported by *in vitro* studies on repeat domain expansion of prion proteins, showing that repeat unit expansion increase aggregation propensity and kinetics of amyloid fibril formation, the latter to such a degree that no lag phase can be observed [33,34]. The absence of a lag phase in fibril formation abolishes the need for a nucleator protein.

### Concluding Remarks

Amyloid marker probes have shown that FuBA can be found within most, if not all, environmental biofilms [6]. Despite the omnipresence of FuBA, very few FuBA systems have so far been characterized and little is known about which bacteria use these fascinating structures and for what purposes. This study has uncovered the phylogenetic diversity of the Fap system. This provides a platform for future studies aiming at determining the function of Fap in various settings. The addition of *fap* deletion strain and *fap* based RT-qPCR assays in animal infection models and in plant growth promoting studies may uncover more on the function of Fap systems. The relatively narrow phylogenetic diversity of the Fap system compared to the curli amyloid systems supports the hypothesis that there may be a range of independently evolved FuBA systems. Consequently, many novel FuBA systems may be discovered across the various microbial phyla. 

## Experimental Procedures

### Identification of Homologous Fap Systems

Fap homologs were initially identified by PSI-Blast searches (default settings, blosum45 scoring matrix, E-value<1) against the refseq protein database using Fap proteins from *P. fluorescens* UK4 as query sequences [35]. The hits were manually curated based on overall proteins primary structure and gene location relative to that of related *fap* gene homologs. Additional homologs were identified by alignment of CDS in the genomic neighborhood of identified fap gene homologs with the previously identified Fap homologs. The curated Fap protein dataset were aligned (ClustalW, gap opening cost (GOC)=15 and gap extension cost (GEC)=1) and used to generate HMMs for the Fap proteins using hmmbuild of the HMMER 3.0 package after removal of redundant proteins. The structural flexibility and low sequence similarity outside the repeat regions of FapB and FapC made confident sequence alignment of the proteins impossible. A combined FapB/C HMM was therefore made in the same way based solely on the repeat regions. The hmmsearch command of the HMMER 3.0 package was used together with the HMMs to search for additional Fap proteins within the refseq protein database. Hits below the inclusion threshold of the HMMs (E-value≈0.01) were discarded. The hits were curated and included in the Fap homolog database and the expanded datasets were used to generate improved HMMs (HMMs S1). This process was repeated until no further homologs could be identified. Identification of Fap homologs within the metagenome databases was done using a similar approach.

### Fap Repeat Identification

Fap repeats were identified by motif search in CLC DNA workbench 5.7.1 (CLC Bio, Aarhus, Denmark) using a java regular expression of the minimalistic Fap repeat (X_15_GX _4_NX _3_GX _6_NX_7_). All repeat regions from bacteria within the same genus were aligned (ClustalW, GOC=50 and GEC=1) in order to determine repeat region consensus sequences.

### Phylogenetic Analysis

16S rRNA gene sequences were obtained for bacterial strains containing homologous Fap systems from the Silva 16S rRNA database (http://www.arb-silva.de/). The 16S rRNA gene sequences were aligned using the SINA v. 1.2.9 aligner (http://www.arb-silva.de/aligner/) and imported to the ARB software [36]. The aligned 16S rRNA genes were used to calculate phylogenetic trees based on the ARB neighbor-joining method provided in the software using the default settings. Homolog Fap protein sequences were aligned (ClustalW, GOC=15 and GEC=1) and imported into ARB. Phylogenetic trees were similarly calculated based on the ARB neighbor-joining method using the default settings. No corrections were applied for among site variation.

## Supporting Information

HMMs S1 **Curated hidden Markov models for the Fap proteins.**
(ZIP)

Table S1 **Fap Protein Homologs Identified within the Refseq Protein Database.** Fap homologs were identified using PSI-Blast searches with the Fap proteins from *P. aeruginosa* PAO1 as query sequences as well as the created HMMs and manual examination of the surrounding gene neighborhoods.
(XLSX)
